# Targeting molecular residual disease and immune escape in colorectal cancer recurrence: biomarker-guided immunotherapy and cell therapies

**DOI:** 10.3389/fmed.2026.1906283

**Published:** 2026-07-22

**Authors:** Yang Shan, Xinyuan Zhang, Jiang Liu, Bin Hu, Lun Yang, Haodi Wang, Jun Li, Zhiwei Jiang, Guanwen Gong

**Affiliations:** 1 The First Clinical Medical College of Nanjing University of Chinese Medicine, Nanjing, Jiangsu, China; 2 Department of General Surgery, Affiliated Hospital of Nanjing University of Chinese Medicine, Nanjing, Jiangsu, China

**Keywords:** CAR-NK, CAR-T, colorectal cancer, ctDNA, immune escape, immunotherapy, molecular residual disease, recurrence

## Abstract

Colorectal cancer (CRC) recurrence after curative-intent treatment remains a major clinical challenge. Conventional postoperative risk stratification relies on clinicopathological variables, whereas recurrence is increasingly understood as a dynamic biological process shaped by molecular residual disease (MRD), immune editing, tumor microenvironmental remodeling and therapy-resistant cellular states. Circulating tumor DNA (ctDNA) has emerged as a clinically relevant MRD biomarker for identifying patients at high risk of relapse and for refining adjuvant-treatment decisions. At the same time, immune biomarkers such as mismatch repair deficiency, microsatellite instability, tumor mutational burden, antigen-presentation status, T-cell exhaustion, myeloid suppression and spatial immune exclusion determine whether recurrent disease is susceptible to immunotherapy. This Mini Review discusses how MRD and immune escape can be integrated into a biomarker-guided framework for recurrent CRC. We summarize recent evidence supporting ctDNA-guided recurrence surveillance, immune checkpoint blockade in MSI-H/dMMR CRC, vaccine-based strategies targeting neoantigens or mutant KRAS, and engineered cell therapies including CAR-T, CAR-NK and TCR-engineered approaches. We also discuss how interpretable machine-learning models and SHAP-based feature attribution may help prioritize recurrence biomarkers and enrich clinical trials, provided that models undergo rigorous external validation. A biomarker-guided strategy linking MRD detection, immune profiling and cell-therapy target selection may provide a translational pathway toward more individualized immune intervention for recurrent CRC. In this framework, AI is positioned as an auditable decision-support layer that links ctDNA kinetics, immune biomarker domains, cell-therapy eligibility, and trial-enrichment decisions rather than as an autonomous treatment selector.

## 1 Introduction

Colorectal cancer (CRC) remains one of the most common malignancies worldwide and a major cause of cancer mortality (1). Although surgery, perioperative chemotherapy, radiotherapy and systemic targeted therapy have improved outcomes, recurrence after curative-intent treatment remains frequent, particularly in stage III disease, locally advanced rectal cancer and resected oligometastatic disease (2–5). The clinical problem is not only whether recurrence can be predicted, but whether recurrence risk can be converted into a rational window for immune-based intervention.

Traditional postoperative recurrence models are mainly based on tumor stage, nodal burden, margin status, lymphovascular invasion, perineural invasion, tumor budding, CEA and treatment-related variables. These factors remain clinically useful, but they do not fully capture the biology of residual tumor cells, immune surveillance and therapy escape. In contrast, the current biomarker landscape increasingly supports a more dynamic view: recurrence reflects a continuum from molecular residual disease (MRD) to clinically detectable relapse, shaped by tumor-intrinsic alterations, immune editing and microenvironmental suppression.

This Mini Review focuses on recurrent CRC through the lens of MRD and immune escape. We discuss ctDNA-defined MRD, immune biomarkers that guide checkpoint blockade and vaccine-based strategies, and biomarker requirements for engineered cell therapies. The review is aligned with the concept that biomarkers should not only estimate prognosis, but also direct immunotherapy and cell-therapy development in clinically actionable subgroups.

## 2 MRD and immune escape as a recurrence continuum

MRD provides a molecular definition of recurrence risk before radiographic relapse. In CRC, postsurgical ctDNA positivity has repeatedly shown strong prognostic value, often outperforming conventional clinicopathological variables in identifying patients likely to relapse (6–10). Large prospective and observational studies have further suggested that ctDNA dynamics may help identify patients who derive benefit from adjuvant chemotherapy and those who may be candidates for de-escalation when ctDNA remains negative (6–9). These findings reposition recurrence surveillance from a schedule-driven process to a biologically informed monitoring strategy.

However, MRD is not merely a marker of microscopic tumor burden. Residual tumor cells must survive immune surveillance, adapt under therapeutic pressure and eventually reconstitute clinically detectable disease. This process involves immune editing, antigen-presentation defects, T-cell dysfunction, myeloid suppression, stromal remodeling and site-specific immune tolerance, particularly in liver metastasis (11–16). Single-cell and spatial studies increasingly show that CRC immune escape is heterogeneous: some tumors are immune-inflamed but exhausted, whereas others are immune-excluded, myeloid-enriched or stromally shielded (12–15).

During the MRD phase, residual CRC cells and the immune system may undergo reciprocal editing before recurrence is visible on imaging. Immune pressure can favor persistence of clones with reduced neoantigen visibility, HLA loss or downregulation, B2M or antigen-processing defects, and impaired interferon-responsive antigen presentation. At the same time, residual tumor cells may promote local immune dysfunction through myeloid recruitment, T-cell exhaustion, suppressive cytokines, stromal remodeling, and liver-metastatic tolerance (15). These changes may arise gradually during the interval between ctDNA positivity and radiographic relapse, creating an immunologically silent period in which molecular disease is detectable but macroscopic tumor architecture has not yet been re-established.

**Figure 1:**
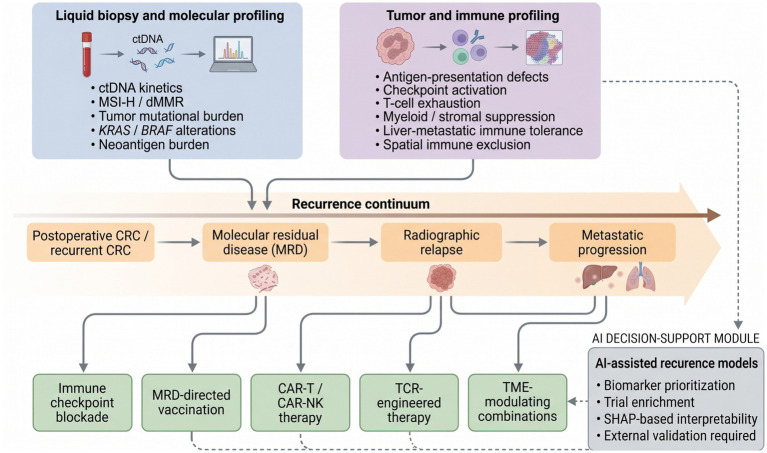
Biomarker-guided workflow for targeting molecular residual disease (MRD) and immune escape in colorectal cancer recurrence. Postoperative or recurrent CRC can be conceptualized as a dynamic continuum from MRD to radiographic relapse and metastatic progression. Liquid biopsy and molecular profiling define recurrence risk through ctDNA kinetics, MSI-H/dMMR status, tumor mutational burden, KRAS/BRAF alterations and neoantigen burden. Tumor and immune profiling define immune escape through antigen-presentation defects, checkpoint activation, T-cell exhaustion, myeloid/stromal suppression, liver-metastatic immune tolerance and spatial immune exclusion. These biomarker layers can guide immune checkpoint blockade, MRD-directed vaccination, CAR-T/CAR-NK therapy, TCR-engineered therapy and TME-modulating combinations. AI-assisted recurrence models and SHAP-based interpretability may support biomarker prioritization and trial enrichment but require external validation.

The central translational implication is that ctDNA-defined MRD should be connected to immune-state profiling. A ctDNA-positive patient with MSI-H/dMMR disease may represent a candidate for checkpoint blockade or perioperative immunotherapy, whereas a ctDNA-positive MSS tumor with myeloid-rich or CAF-dominant exclusion may require TME-modulating combinations. Similarly, MRD carrying actionable neoantigens, mutant KRAS peptides or persistent lineage antigens may be suitable for vaccine or engineered cell-therapy strategies. Together, these recurrence-associated molecular, antigenic and microenvironmental features support a layered biomarker workflow for immune-based treatment selection in recurrent CRC (Figure 1).

## 3 ctDNA and molecular biomarkers for recurrence-directed intervention

ctDNA is currently the most mature MRD biomarker in CRC. Randomized and prospective studies have demonstrated that ctDNA-guided approaches can reduce unnecessary adjuvant chemotherapy in selected stage II colon cancer while maintaining favorable recurrence-free outcomes (6, 9). Large-scale analyses from the CIRCULATE-Japan platform further support postsurgical ctDNA as a strong prognostic marker and as a tool for identifying patients who may benefit from adjuvant chemotherapy (7, 8). Meta-analytic evidence also confirms that ctDNA detection after curative-intent surgery is associated with substantially increased recurrence risk (10).

In recurrent or oligometastatic CRC, ctDNA may also define residual disease after metastasectomy or local therapy (17–20). This is clinically relevant because radiographic imaging may miss early molecular relapse, and CEA lacks sufficient sensitivity and specificity for individualized immune intervention. Serial ctDNA kinetics can capture molecular progression, treatment response and emergence of resistant clones. KRAS, NRAS, BRAF, HER2 amplification, NTRK fusions and other genomic alterations may also inform targeted or combination strategies, although most are not direct immunotherapy biomarkers.

The key challenge is to translate ctDNA positivity into immune-based action. At present, ctDNA is best validated as a recurrence-risk biomarker rather than a standalone indication for immunotherapy. Future trials should test whether MRD-positive patients can be enriched for immune interventions based on additional features, including MSI-H/dMMR status, neoantigen burden, HLA genotype, antigen-presentation integrity, interferon signaling and immune-cell composition. In this setting, ctDNA may define when to intervene, while immune and antigen biomarkers define how to intervene.

From a decision-support perspective, serial ctDNA results can be treated as time-updated inputs rather than static variables. When combined with MSI/MMR status, HLA and antigen-presentation features, immune-infiltration signatures, spatial immune-exclusion patterns, and planned cell-therapy targets, these data can feed interpretable AI models that triage patients for surveillance intensity, MRD-enriched trials, re-biopsy, vaccine design, or cell-therapy eligibility.

## 4 Biomarkers guiding checkpoint blockade and vaccine-based strategies

MSI-H/dMMR status remains the strongest clinically established immunotherapy biomarker in CRC. In metastatic MSI-H/dMMR CRC, pembrolizumab improved outcomes compared with chemotherapy in the first-line setting (21). Neoadjuvant PD-1 blockade has produced major responses in dMMR rectal cancer, and combination checkpoint blockade in nonmetastatic dMMR colon cancer has shown striking pathological responses (22, 23). More recent efforts incorporating dual checkpoint strategies such as PD-1 and LAG-3 blockade further support the relevance of immune-state biomarkers in localized dMMR colon cancer (24).

The problem is that most recurrent CRC is microsatellite-stable (MSS), where checkpoint blockade alone is usually ineffective (11). MSS recurrence is commonly characterized by lower neoantigen immunogenicity, impaired T-cell infiltration, myeloid-driven suppression, T-cell exhaustion and stromal exclusion (11–16). Therefore, biomarker-guided immunotherapy in recurrent CRC must move beyond MSI status. Candidate biomarkers include TMB, neoantigen quality, HLA loss, B2M alteration, interferon pathway status, PD-L1 expression, CD8 + T-cell infiltration, exhaustion markers, macrophage polarization, CAF signatures and liver-metastatic immune suppression (12–16, 25–27).

Vaccine-based strategies are particularly relevant to MRD because the disease burden is lower and immune suppression may be less entrenched than in bulky metastatic disease. Neoantigen-directed vaccines and mutant KRAS-targeted vaccines provide a conceptual bridge between molecular relapse and immune intervention (28–30). Early-phase studies of lymph-node-targeted mutant KRAS vaccines in pancreatic and colorectal cancer suggest that antigen-specific immune activation is feasible, including in patients with minimal residual mutant KRAS disease (28, 29). For recurrent CRC, the most rational vaccine trials will likely combine ctDNA-defined MRD, HLA-restricted neoantigen selection, immune monitoring and rational checkpoint or TME-modulating combinations.

Several ongoing or recently reported trial programs illustrate this shift toward prospective MRD-guided intervention. The CIRCULATE-Japan platform links the GALAXY observational registry with interventional escalation or de-escalation studies such as ALTAIR and VEGA, whereas CIRCULATE-North America/NRG-GI008 evaluates adjuvant chemotherapy strategies according to postoperative ctDNA-defined residual disease. DYNAMIC-III further supports the feasibility of ctDNA-guided adjuvant treatment in locally advanced colon cancer. For immune-directed strategies, KRAS-directed vaccine studies, MRD-positive vaccine trials, and early CAR-T/CAR-NK or TCR-engineered studies increasingly use mutation, HLA, antigen-expression, and ctDNA criteria for enrollment. These trials should be discussed as evolving platforms rather than definitive evidence that ctDNA positivity alone is sufficient to select immunotherapy (31–34).

## 5 Cell therapies in recurrent CRC: target selection and immune constraints

Engineered cell therapies are increasingly being explored in CRC, but their success depends on precise biomarker-defined target selection. Unlike hematologic malignancies, CRC presents several barriers to cell therapy, including heterogeneous antigen expression, on-target off-tumor toxicity risk, limited trafficking, stromal exclusion, hypoxia, immunosuppressive cytokines, myeloid cells and exhaustion of infused cells (35–42). These barriers make recurrent CRC a model disease in which cell therapy must be developed together with biomarker-defined patient selection.

CAR-T strategies in CRC have focused on surface targets such as CEA, GCC/GUCY2C, HER2, EpCAM, MUC1 and other tumor-associated antigens. Recent clinical data for GCC-targeted CAR-T cells demonstrate that objective activity in heavily pretreated metastatic CRC is possible, although durability, toxicity, antigen heterogeneity and optimal combination strategies remain unsettled (35–37). These findings indicate that recurrence and metastatic disease should not be viewed as immunologically inaccessible, but they also show that antigen expression alone is insufficient. A clinically useful CAR-T biomarker framework should incorporate target density, tumor distribution, normal-tissue expression, soluble antigen burden, trafficking markers and TME suppression.

Clinically accessible target assessment should combine several imperfect tools. Immunohistochemistry can quantify antigen-positive tumor-cell fraction and staining intensity, but sampling bias and intratumoral heterogeneity remain major limitations. Multiplex immunohistochemistry or spatial profiling can relate target expression to stromal barriers and immune exclusion, whereas molecular imaging or antigen-directed PET, when available, may provide lesion-level assessment of whole-body target distribution. Liquid biopsy can monitor molecular burden and emerging resistance but generally cannot directly measure surface-protein density (38). Therefore, treatment decisions should use prespecified antigen thresholds, such as minimum target-positive tumor-cell fraction, intensity score, lesion concordance, and tumor-to-normal expression ratio, rather than simple positive/negative classification.

CAR-NK approaches may offer complementary advantages, including innate cytotoxicity, potential allogeneic use and lower risk of severe cytokine release syndrome, although persistence and expansion remain challenges (38–42). CEA-CAR-NK-92 and NKG2D CAR-NK platforms are being explored for solid tumors and advanced CRC (39–42). NKG2D-based approaches are attractive because stress ligands may be induced on malignant cells, but the same biology raises questions about ligand heterogeneity, immune escape and safety. TCR-engineered therapy and TIL-based strategies may be more suitable for intracellular neoantigens or viral antigens, but require HLA restriction, antigen-presentation competence and careful selection of clonotypes. Across these modalities, MRD may represent a more favorable intervention window than bulky recurrent disease because antigen burden, immune suppression and physical exclusion may be less severe.

## 6 AI-assisted clinical decision support and biomarker prioritization

Machine-learning models, nomograms and explainable AI can support biomarker-driven recurrence management, but they should not become a substitute for biological validation. Recent CRC studies show that machine-learning survival models can integrate clinicopathological and molecular variables to predict recurrence or survival, and SHAP analysis can help identify variables contributing to individualized risk (43–46). This is relevant to the present topic because interpretable models may help prioritize which MRD-positive patients should enter immune-intervention trials and which biomarker domains should be measured prospectively.

For example, a recurrence model may identify lymph node ratio, tumor budding, R0 status, perineural invasion, ctDNA status, MSI-H/dMMR, KRAS/BRAF alterations or immune-infiltration variables as risk drivers. SHAP-based explanations can make these contributions visible and may help translate statistical risk into trial-enrichment hypotheses. Nevertheless, AI models are vulnerable to retrospective bias, missing variables, center effects, data leakage, calibration failure and poor transportability. Reporting and validation frameworks such as DECIDE-AI and TRIPOD+AI emphasize prospective evaluation, transparent reporting, clinical workflow integration and external validation before clinical deployment (47, 48). In recurrent CRC, AI should therefore be used to organize biomarker complexity and generate testable strategies, not to independently assign immunotherapy or cell therapy outside validated protocols.

Important limitations must be addressed before any AI-supported workflow is used clinically. These include non-standardized ctDNA assays, incomplete longitudinal sampling, missing pathology or imaging variables, retrospective training bias, center effects, class imbalance, data leakage, poor calibration, limited interpretability, uncertain benefit in external populations, and ethical concerns regarding automated prioritization. Therefore, AI outputs should be reported with uncertainty, audited for subgroup performance, and evaluated prospectively before they guide treatment selection.

## 7 Future perspectives

Future development should move beyond single-marker enrichment toward multidimensional recurrence assessment. Transcriptomic and spatial-transcriptomic profiling can define immune-excluded, myeloid-enriched, CAF-dominant, and interferon-active recurrence states; single-cell sequencing can resolve resistant epithelial, myeloid, T-cell, NK-cell, and stromal subpopulations; and digital pathology can provide scalable whole-slide assessment of immune architecture, tumor budding, stromal exclusion, and metastatic-site heterogeneity. These modalities should be connected to serial ctDNA monitoring so that molecular relapse, tissue context, and immune accessibility are interpreted together (13, 14, 47, 48).

Adaptive clinical trials represent the most appropriate testing environment for this framework. MRD-positive patients could be stratified by MSI/MMR status, neoantigen or KRAS vaccine eligibility, target-antigen expression, HLA-restricted TCR eligibility, and suppressive TME features, with predefined adaptive rules for treatment escalation, de-escalation, re-biopsy, or combination therapy. Future studies should report not only response or recurrence-free survival, but also ctDNA clearance, immune-cell expansion, antigen loss, spatial immune remodeling, toxicity, quality of life, and feasibility of implementing AI-assisted decision support across centers.

## 8 Discussion and conclusion

Recurrent CRC should increasingly be understood as a biomarker-defined state rather than a uniform clinical event. MRD detection identifies when relapse risk becomes biologically visible, whereas immune profiling helps define whether residual or recurrent disease is susceptible to checkpoint blockade, vaccine-based intervention, engineered cell therapy or TME-modulating combinations. This distinction is important because ctDNA positivity alone does not specify the optimal treatment; it defines a molecular window that must be interpreted alongside tumor antigenicity, immune contexture and therapeutic targetability.

The strongest current evidence supports ctDNA as a prognostic and treatment-stratification biomarker after curative-intent treatment (6–8). For immune intervention, MSI-H/dMMR status remains the most clinically actionable biomarker, but this subgroup represents only a minority of CRC recurrence (21–23). The larger clinical challenge lies in MSS recurrent disease, where immune escape is driven by a combination of low immunogenicity, stromal exclusion, suppressive myeloid biology, exhaustion and metastatic-organ immune tolerance (11–14). For this reason, future biomarker-guided trials should not treat recurrent CRC as a single category. Instead, they should define MRD-positive, MSI-H/dMMR, neoantigen-rich, target-antigen-positive, myeloid-enriched, CAF-dominant or immune-excluded subgroups.

Cell therapies provide an additional layer of opportunity but also require stricter biomarker logic. For CAR-T and CAR-NK therapies, target expression must be paired with evidence of therapeutic accessibility, safety, trafficking and resistance mechanisms (35–38). For TCR-engineered or neoantigen-directed approaches, HLA restriction and antigen-presentation competence are essential. The most plausible near-term strategy may be to combine MRD-guided timing with antigen-guided targeting and TME-guided combinations. Such an approach could support smaller, biomarker-enriched trials rather than broad unselected trials in heavily pretreated metastatic CRC.

Several limitations should be acknowledged. First, ctDNA assays differ in sensitivity, tumor-informed design, tumor-naive design and sampling schedule, which affects clinical interpretation. Second, immune biomarkers are highly spatial and dynamic; a primary tumor specimen may not reflect recurrent or metastatic disease. Third, many cell-therapy studies in CRC remain early phase, with limited patient numbers and heterogeneous target criteria. Fourth, AI-assisted recurrence models often lack prospective external validation and may not generalize across populations or clinical workflows (46–48).

In summary, targeting MRD and immune escape offers a rational framework for biomarker-guided immunotherapy and cell therapies in recurrent CRC. The field should move from recurrence prediction alone toward recurrence-directed immune intervention. This will require prospective MRD-integrated trials, harmonized immune profiling, validated antigen-selection criteria, transparent AI-supported risk models and clinically meaningful endpoints such as recurrence-free survival, durable response and treatment-related toxicity. If these requirements are met, recurrent CRC may become a testable model for converting molecular relapse into personalized immune-based treatment. The next phase should integrate transcriptomics, spatial and single-cell technologies, digital pathology, and adaptive trial design into a unified MRD-to-immunotherapy pipeline (Table 1).

**Table 1:** Biomarker modules for immune intervention in recurrent colorectal cancer.

Biomarker module	Representative biomarkers	Relevance to recurrence	Implication for immunotherapy or cell therapy
MRD and liquid biopsy	ctDNA positivity, ctDNA kinetics, CEA, tumor-informed or plasma-only ctDNA assays	Defines molecular relapse risk before radiographic recurrence	Identifies intervention window and supports MRD-enriched trials
Tumor molecular profile	MSI-H/dMMR, TMB, KRAS/NRAS/BRAF, HER2, NTRK, neoantigen burden	Separates immunogenic, targetable and resistant recurrence states	Guides checkpoint blockade, vaccines, targeted combinations and trial enrollment
Antigen presentation	HLA genotype, B2M, TAP1/TAP2, IFN-response genes	Determines immune recognition and risk of immune escape	Required for TCR-engineered therapy and neoantigen vaccine strategies
Tumor surface targets	GCC/GUCY2C, CEA, HER2, EpCAM, MUC1, NKG2D ligands	Defines targetability of recurrent or metastatic deposits	Supports CAR-T or CAR-NK target selection and safety assessment
Immune contexture	CD8 + T cells, GZMB, PRF1, PD-1/PD-L1, LAG-3, TIGIT, T-cell exhaustion	Distinguishes inflamed, exhausted and immune-cold recurrence	Guides checkpoint blockade and combination immunotherapy
Suppressive TME	TAM, MDSC, CAFs, TGF-beta, hypoxia, liver-metastatic immune tolerance	Promotes immune exclusion and resistance in MSS recurrent CRC	Supports myeloid, stromal or metabolic combination strategies
Target assessment and heterogeneity	IHC intensity, antigen-positive fraction, lesion concordance, target-to-normal expression ratio, antigen-directed PET where available, soluble antigen burden	Determines whether target expression is sufficient, spatially accessible, and stable across lesions	Supports threshold-based CAR-T/CAR-NK eligibility, dual-target design, and monitoring for antigen-negative escape
